# High-risk human papillomaviruses and Epstein–Barr virus in breast cancer in Lebanese women and their association with tumor grade: a molecular and tissue microarray study

**DOI:** 10.1186/s12935-021-02009-4

**Published:** 2021-06-10

**Authors:** Karim Nagi, Ishita Gupta, Nawaf Jurdi, Ayesha Jabeen, Amber Yasmeen, Gerald Batist, Semir Vranic, Ala-Eddin Al-Moustafa

**Affiliations:** 1 College of Medicine, QU Health, Qatar University, PO Box 2713, Doha, Qatar; 2 Biomedical and Pharmaceutical Research Unit, QU Health, Qatar University, PO Box 2713, Doha, Qatar; 3 Biomedical Research Centre, QU Health, Qatar University, PO Box 2713, Doha, Qatar; 4 Department of Pathology and Laboratory Medicine, American University of Beirut Medical Center, Beirut, Lebanon; 5 Segal Cancer Centre, Lady Davis Institute for Medical Research/JGH, McGill University, Montreal, QC H3A 0G4 Canada; 6 Oncology Department, McGill University, Montreal, QC H3A 0G4 Canada

**Keywords:** HPV, EBV, Breast cancer, Tumor grade, Lebanese population

## Abstract

**Background:**

High-risk human papillomaviruses (HPVs) are present and can cooperate with Epstein–Barr virus (EBV) to initiate and/or enhance the progression of several types of human carcinomas including cervical as well as head and neck; in parallel, it has been recently pointed out that these oncoviruses can be detected in human breast cancers. Thus, we herein explored the presence/co-presence of high-risk HPVs and EBV in breast cancer in Lebanese women.

**Methods:**

A cohort of 102 breast cancer samples and 14 normal breast tissues were assessed for the presence of HPVs and EBV. Polymerase chain reaction (PCR) and immunohistochemistry (IHC) analysis in addition to tissue microarray (TMA) platform were used in this study.

**Results:**

We found the presence of HPV in 66/102 (65%) of our samples, while EBV is present in 41/102 (40%) of the cohort. Additionally, our data showed that high-risk HPV types (52, 35, 58, 45, 16 and 51) are the most frequent in breast cancer in Lebanese women. Meanwhile, we report that high-risk HPVs and EBV are co-present in 30/102 (29%) of the samples; more significantly, our results indicate that their co-presence is associated with tumor grade (*p* = 0.03).

**Conclusion:**

Our data revealed that HPVs and EBV are present/co-present in human breast cancer where they may play an important role in its development and/or progression; thus, we believe that further investigations are essential to confirm and elucidate the presence/co-presence of these oncoviruses and the underlying mechanisms of their interaction in breast carcinogenesis.

## Background

Breast cancer is the leading cause of cancer-related death in the worldwide female population, comprising 25% of all cancer cases [1]. Incidence of this disease is continuously increasing both in developed and developing countries. Similarly in Lebanon, a Middle Eastern country, breast cancer is the most frequent cancer among women, with the highest rate in the region comprising around 38% of the cases [2]. While breast cancer mortality rates vary greatly worldwide, ranging from 20% or below in North America, to at least 40% in low- and middle-income countries [3]. In fact, the majority of breast cancer deaths are the result of metastasis in woman presenting for diagnosis at later stages of the disease with high invasiveness, lymph node involvement and more aggressive forms [4, 5].

Human papillomavirus (HPV), a non-enveloped double-stranded DNA virus is transmitted sexually [6, 7]. The HPV genome consists of eight open reading frames (ORFs) and is separated into three functional elements; the early (E) region which encodes six proteins (E1, E2, E4–E7) and is involved in regulating viral transcription and replication, the late region that encodes the structural proteins (L1 and L2) and is involved in viral assembly, and, the long control region [8]. On the other hand, Epstein-Barr virus (EBV) is a double-stranded DNA gamma-herpes virus [9, 10]. The EBV genome spans 184-kbp and encodes around 85 genes including the six EBV-encoded nuclear antigens (EBNA1, -2, -3A, -3B, -3C and -LP) and latent membrane proteins (LMP1, -2A, and -2B), as well as various noncoding RNAs (EBERs and miRNAs) [10, 11]. Along with the presence of other oncogenes and/or oncoviruses, these agents can infect epithelial cells (epidermal or mucosal) and their proteins impair cell cycle progression thus inducing neoplastic transformation [6, 12, 13]. In this context, in cancers associated with lymph node metastases and vascular invasion such as cervical, HN and colorectal cancers, the E6 and E7 oncoproteins of high-risk HPVs are constitutively expressed and play a role in inactivating tumor suppressors [14]. HPVs immortalize and change the proliferative properties of human mammary epithelial cells [15, 16]. More precisely, while HPV E6 oncogenic proteins facilitate the degradation of p53 [17], E7 proteins of high-risk HPVs mediate the increase of p16 assembly [18] and bind to retinoblastoma [19, 20], as well as to other pocket proteins, such as p107 and p130 [21], leading to cell cycle deregulation. In this respect, we have previously reported that E6 and E7 oncoproteins of high-risk HPV type 16 can induce genomic instability and help converting non-invasive and non-metastatic breast cancer cells into invasive and metastatic phenotypes [22]. Similarly to high-risk HPV, it has been revealed that EBV infection can be associated with several types of human carcinomas including nasopharyngeal, cervical, gastric in addition to breast [13]. Indeed, oncoproteins of EBV (especially LMP1 and EBNA1) can enhance cell proliferation and motility in addition to angiogenesis, while inhibiting apoptosis, which are major events in cancer progression [23–25].

Interestingly, high-risk HPVs types 18, 16 and/or 33 have been detected in breast cancer tissues of woman from the Middle-East [26–29]. However, a recent study showed that HPV types 16 and 33 were not detected in breast cancer samples from Qatari women showing that these viral infections in the Middle-East could be linked to specific geographic locations. In addition, recent investigations revealed that high-risk HPVs and EBV can be co-present in several types of human carcinomas including HN, colorectal, cervical and breast; and their co-presence is associated with highly aggressive tumor phenotypes [30–35]. However, no study thus far regarding this important topic was reported in Lebanon. Accordingly, we herein explored the presence/co-presence of high-risk HPVs and EBV in breast cancer samples from Lebanese women by PCR and tissue microarray (TMA) analysis. Our data revealed that co-presence of both HPV and EBV was found in 29% of the examined samples and their co-presence is significantly associated with tumor grade.

## Materials and methods

### Sample collection and DNA extraction

Primary and naïve breast cancer samples from a total of 116 Lebanese female patients were collected over a 10-year period (2006–2016). All samples and histopathology reports were de-identified and data were analyzed anonymously. In accordance with the ethical standards of the Lebanese legislation, practicing physicians orally informed all breast cancer patients about potential utilization of biopsies for research or secondary purpose. Therefore, a written informed consent was not required. Additionally, the Institutional Biosafety Committee (IBC) approval was obtained from Qatar University (QU-IBC-2018/22) to explore the presence of HPV and EBV in human breast cancers.

All the tumors were graded according to the Nottingham histological grade (modified Scarff-Bloom-Richardson grade) [36]. The tumors were considered positive for estrogen (ER) and progesterone receptors (PR) if nuclear positivity was observed in > 1% of tumor cells [37]. For HER2 expression, positivity (score 3+) was defined as intense, complete, circumferential membranous expression in > 10% tumor cells [38], while equivocal (score 2+) was defined as weak, complete membranous staining in > 10% of tumor cells. Tumor cells with scores 0–1+ were considered negative for HER2. The proliferation index was assessed using Ki-67 proliferation marker (MIB1 antibody). Tumors were considered highly proliferative if Ki-67 labeling was observed in > 20% of tumor cells.

All samples (punch samples of 2 mm thickness) were taken from the formalin-fixed paraffin embedded (FFPE) blocks of surgically removed and pathologically confirmed invasive breast carcinomas. Exclusion criteria encompassed patients treated with neoadjuvant therapy (chemotherapy, endocrine therapy, radiotherapy, HER2-based or any other targeted therapies), and patients of other nationalities in addition to Lebanese male patients with breast cancer.

DNA extraction from FFPE tissues was performed using the Thermo Scientific GeneJET FFPE DNA Purification Kit (ThermoFisher Scientific, USA) as previously described [35]. In brief, FFPE sections were digested using 200 µl of the digestion buffer and 20 µl of the Proteinase K solution. The released genomic DNA was then de-crosslinked by heat incubation at 90 °C for 40 min. The resulting solution was further centrifuged and the supernatant containing DNA was mixed with 200 µl of the binding buffer. Ethanol (96%) was then added and the lysate loaded onto the purification column. Finally, the adsorbed DNA was subjected to washing to remove contaminants before being eluted with 60 µl of the elution buffer.

### HPV and EBV detection by PCR

Genotyping and detection of the presence of HPV and EBV was done using primers specific for high-risk HPV types (16, 18, 31, 33, 35, 39, 45, 51, 52, 56, 58, 59, 66 and 68) of the E6/E7 region and for EBV genes, EBNA1 and LMP1 as previously described [39, 40]. GAPDH was used as an internal control. Analysis was performed as previously described by our group [39, 40].

PCR was performed using the Invitrogen Platinum II Hot-Start Green PCR Master Mix (2X) (ThermoFisher Scientific, USA) as described previously [35, 39, 40]. HPV and EBV genes were amplified for an initial denaturation at 94 °C for 2 min followed by 40 cycles of 94 °C for 30 s, annealing at temperatures ranging from 50 to 62 °C for 30 s depending on each primer’s melting temperature as previously described [39, 40], and 72 °C for 30 s with a final incubation of 10 min at 72 °C. The PCR product from each exon was resolved using 1.5% agarose gel electrophoresis and visualized using iBrightCL1000 Imaging System (ThermoFisher Scientific, USA). In each experiment, negative control (instead of DNA, MDA-MB-453 cell line [41] and sterile water) and positive control (Hela cell line for L1 region [42] and normal oral epithelial (NOE) cell line expressing E6/E7 of HPV type 16 for E6/E7 region [43]) were used.

### Tissue microarray (TMA)

Tissue microarray construction was performed as described previously by our group [29, 32, 34]. In brief, both, control and cancer samples were embedded into virgin paraffin TMA blocks using a manual tissue arrayer (Beecher Instruments, Silver Spring, MD, USA). All FFPE samples were de-identified and assembled without any prior knowledge of linked clinical or pathological staging information.

Two TMA cores of 1.0 mm in diameter were sampled from a cohort of 116 FFPE samples (14 control and 102 cancer samples) from Lebanese patients. Next, sections of 4 µm were cut and stained with hematoxylin and eosin (H&E) on the initial slides to verify the histopathologic diagnosis for cancer tissues. Afterwards, H&E staining was performed, slides of the completed blocks were used for immunohistochemistry (IHC) assays (against E6 and LMP1 of high-risk HPV and EBV, respectively).

### Immunohistochemistry (IHC) analysis

To further confirm the expression of E6 and LMP1 oncoproteins of HPV and EBV, respectively, immunohistochemical analysis was carried out using previously described methodology [29]. To analyze protein expression patterns of E6 and LMP1 in TMA slides, each slide was subjected to deparaffinization in gradient alcohol, rehydrated and boiled (microwave) in 10 mM citrate sodium citrate solution (pH 6.0) for 10 min. This was followed by blocking endogeneous peroxidase activity using 3% hydrogen peroxide in methanol, TMA slides were further incubated for 35 min at 37 °C with primary monoclonal antibodies for E6 of HPV (clones 1–4 and C1P5, Dako Agilent, Carpinteria, CA and Calbiochem, Canada) and LMP1 of EBV (clone CS1–4, Abcam) using a fully automated immunostainer (Ventana Medical System, Tuscon, AZ). The fully automated Ventana Medical System uses an indirect biotin–avidin system with a universal biotinylated immunoglobulin secondary antibody. Prior to mounting, slides were counterstained with hematoxylin and staining procedures were completed as per the manufacturer’s recommendations. Negative controls were obtained by omitting specific primary antibody for E6 and LMP1.

Tumors exhibiting positivity ≥ 5% of the cells were considered positive for E6 and LMP1 [33]. LMP1 protein expression (EBV) was further assessed for the expression in tumor-infiltrating lymphocytes and stromal cells [33].

### Statistical analysis

Statistical analysis was performed using IBM Statistical Package for the Social Sciences (SPSS, version 25). To assess the significance of HPV and EBV association as well as between clinicopathological data (patient’s age and Nottingham histological grade) in correlation with the presence/co-presence of HPVs and EBV, we utilized Chi-square (χ^2^) test with Yates’ correction and Fisher’s exact test. Graphs were plotted using GraphPad Prism software (version 8.4.3). Data were calculated as non-parametric and statistical significance was achieved at p < 0.05.

## Results

### Clinicopathological characteristics of the cohort

The clinicopathological characteristics of the cohort are summarized in Table 1. The mean age of all patients was 52.4 (standard deviation (SD), ± 13.7) years. Most of the patients (51%) are aged > 50 years. Axillary lymph node status was available for 94 cases of which 64% had positive axillary lymph nodes (Table 1). The Nottingham histological grade was available for 99 of our cancer samples; the majority of the cases (64%) are grade III breast cancer followed by grade II (30%) and grade I (6%) (Table 1).

**Table 1:** Clinicopathological characteristics of patients with breast cancer

Characteristic	Categories	Number (%)
Age	≤ 50	50 (49)
	> 50	52 (51)
Histopathological Subtypes of Breast Cancer	Invasive Ductal Carcinoma (IDC)	92 (90.2)
	Invasive Lobular Carcinoma (ILC)	6 (5.9)
	Mixed Carcinoma (IDC + ILC)	4 (3.9)
Nottingham Histological Grade	I	6 (5.8)
	II	31 (30.4)
	III	65 (63.8)
Tumor Stage (pT)	pT1	9 (8.8)
	pT2	73 (71.6)
	pT3	13 (12.7)
	pT4	7 (6.9)
Lymph Node Involvement (pN)	pN0	29 (28.4)
	pN1	31 (30.4)
	pN2	19 (18.6)
	pN3	15 (14.7)
	Unknown	8 (7.9)
Estrogen Receptor (ER) Status	ER+	79 (77.5)
	ER-	20 (19.6)
	Unknown	3 (2.9)
Progesterone Receptor (PR) Status	PR+	66 (64.7)
	PR−	33 (32.4)
	Unknown	3 (2.9)
HER2 Status	Positive (3+)	8 (7.9)
	Equivocal (2+)	22 (21.6)
	Negative (0–1+)	69 (67.6)
	Unknown	3 (2.9)
Ki-67 Proliferative Index (PI)	Low (< 10%) PI	16 (15.7)
	Intermediate (10–20%) PI	12 (11.8)
	High (> 20%) PI	25 (24.5)
	Unknown	49 (48)
HPV Expression	Invasive ductal carcinoma (IDC)	61 (59.8)
	Invasive lobular carcinoma (ILC)	3 (2.9)
	Mixed carcinoma (IDC + ILC)	1 (0.9)
	Invasive micropapillary carcinoma	1 (0.9)
EBV Expression	Invasive ductal carcinoma (IDC)	33 (32.4)
	Invasive lobular carcinoma (ILC)	5 (4.9)
	Mixed carcinoma (IDC + ILC)	2 (1.9)
	Invasive micropapillary carcinoma	1 (0.9)

The majority of cancer cases were invasive ductal carcinomas (IDC), no special type (NST) (95 cases, 93%), while 7 cases (7%) were invasive lobular carcinoma (ILC). The hormone receptor status was available for 99 of the cases; 79 (77%) and 66 (65%) were positive for estrogen and progesterone receptors, respectively. With reference to HER2 expression, its status was available for 99 cases of which 8 (8%) overexpress HER2 (score 3+), 22 (21%) had equivocal expression (score 2+), and 69 (68%) lacked HER2 expression (scores 0–1+). Confirmatory *in-situ* hybridization assessment of HER2 equivocal (2+) cases was not available.

The status for Ki-67 proliferative index was also available for 53 cases, 16 of which had a low proliferation rate (< 10%), 12 were intermediate (10–20%) and the remaining 25 cases had a high (> 20%) proliferation rate (Table 1).

### Status of high-risk HPV subtypes and EBV by PCR

**Fig. 1:**
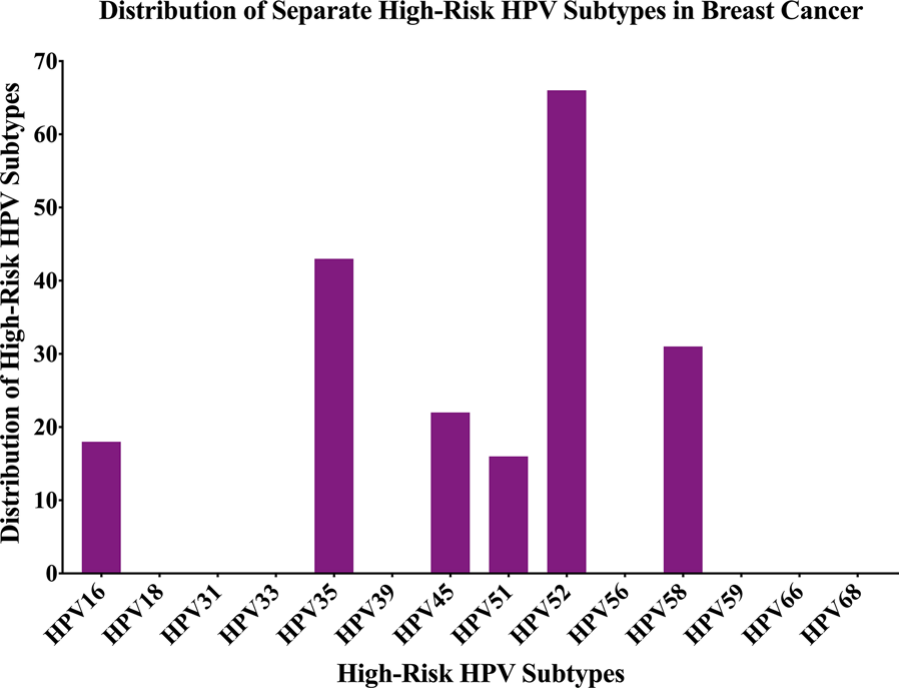
The distribution of each high-risk HPV subtype in Lebanese breast cancer samples. The PCR analysis included 102 breast cancer samples revealing that the most frequent HPV subtypes are 52, 35, 58, 45, 16 and 51

We found that 66 of the 102 samples were positive for high-risk HPVs (64.7%); and the most commonly present high-risk HPV was HPV52 (65%), followed by HPV35 (42%), HPV58 (30%), HPV45 (22%), HPV16 (18%) and HPV51 (16%) (Fig. 1). HPV types 18, 31, 33, 39, 56, 59, 66 and 68 were not detected in our examined samples (Fig. 1).

On the other hand, we noted that 41/102 (40.2%) of the samples were positive for EBV; among those 41 cases, EBNA1 and LMP1 were individually present in 41/41 (100%) and 36/41 (88%) of the cases, respectively. Additionally, while some normal breast samples revealed positivity for high-risk HPVs (5/14, 35.6%), EBV was absent in all normal breast samples (0/14, 0%). However, it is important to highlight that there was a significant difference in HPV-positivity between normal and tumor breast tissue samples (*p* = 0.04).

Furthermore, our data pointed out that the most common co-infections of breast cancer samples were with HPV52 and other high-risk HPV subtypes. Three or more co-infections were seen in 30/102 (29%) breast cancer samples, 43 cases (42.1%) have three HPV co-infections, 16 cases (15.7%) have four co-infections and 5 cases (5%) have five high-risk HPV co-infections.

More significantly, our data revealed that high-risk HPVs and EBV are co-present in 29% (30/102) of breast cancer cases (Table 2). In addition, a significant correlation between EBV and HPV types, HPV52 (*p* = 0.03) and HPV58 (*p* = 0.002) was found in our cohort of breast cancer samples (χ2 test with Yates’ correction) (Table 2).

**Table 2:** Correlation of EBV and HPV-subtypes in Lebanese breast cancer patients

Samples	No. of cases	High-risk HPV types					
		16	35	45	51	52	58
EBV (+)	41	5	17	7	5	32	20
EBV (−)	61	13	26	15	11	34	11
Total	**102**	**18**	**43**	**22**	**16**	**66**	**31**
*p-value*		**0.35**	**0.92**	**0.51**	**0.60**	0.03*	0.002**

* Indicates significant p-values (*p < 0.05, **p < 0.01)

N/A denotes Not Applicable as χ^2^ test is invalid in these cases, since value is 0

### Expression patterns of E6 and LMP1 of high-risk HPVs and EBV

**Fig. 2:**
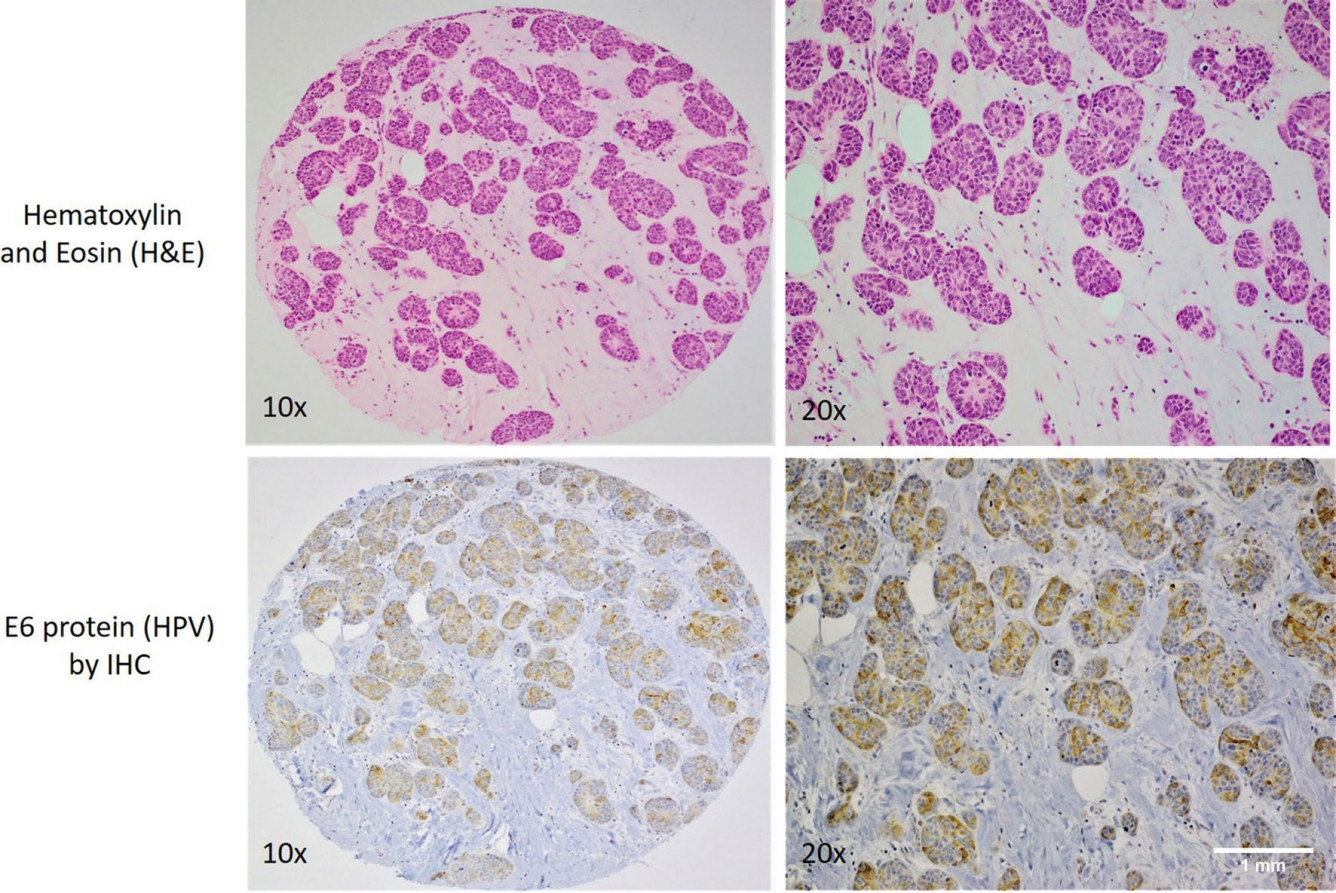
A case of invasive ductal carcinoma (upper images, Hematoxylin and Eosin stain) with E6 protein (HPV) expression by immunohistochemistry (lower images)

Among the 116 samples used for IHC to detect E6 (high-risk HPV) and LMP1 (EBV), thirty-seven of the TMA blocks did not contain cancerous tissues, which could be likely due to a lack of exact H&E/FFPE matching required for normal TMA construction. Fifty of the remaining seventy-nine cases (63.3%) revealed positive for E6 of HPV positivity in these samples by IHC above the threshold of 1% of positive cancer cells. The positivity of E6 oncoprotein of high-risk HPV was predominant in cancer cells (Fig. 2) and E6 was localized in both cytoplasmic and nuclear compartments. The remaining cases were negative for E6 of HPV. In addition, the E6 expression was sporadically observed in adjacent benign and normal ducts as well as non-invasive breast carcinomas (ductal carcinoma in situ/DCIS).

**Fig. 3:**
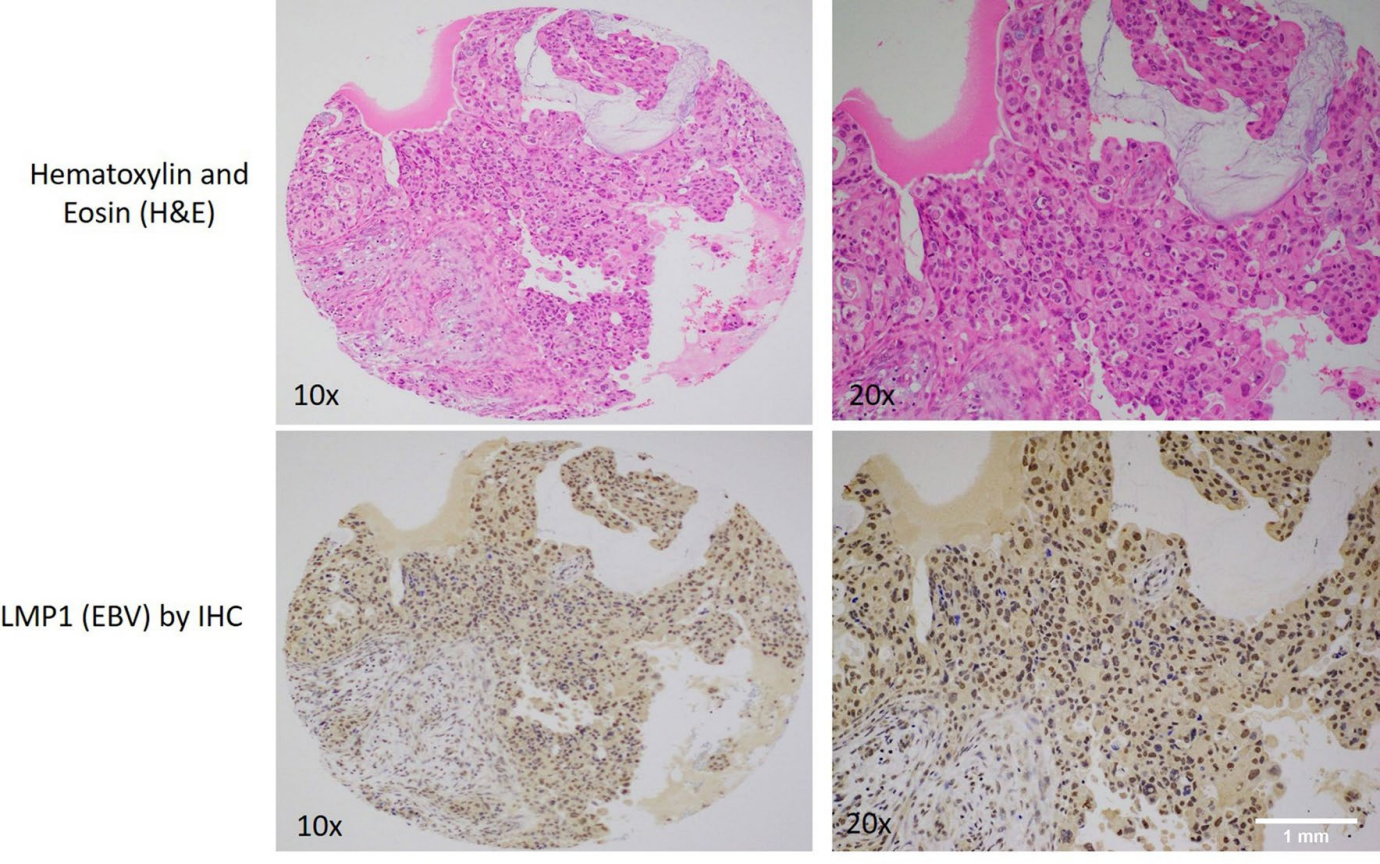
A case of invasive breast carcinoma with apocrine differentiation and focal mucin production (upper images, Hematoxylin and Eosin stain) with a LMP1 protein (EBV) positivity by immunohistochemistry (lower images)

In the same seventy-nine TMA blocks, LMP1 oncoprotein of EBV was positive in thirty-one of the seventy-nine cancer cases (39.2%) and its localization was predominantly cytoplasmic and nuclear (Fig. 3); occasionally, LMP1 was expressed in benign and normal ducts as well as adjacent stromal lymphocytes (tumor infiltrating lymphocytes).

Twenty-three of the 79 samples (29.1%) were positive (≥ 1% positive cancer cells) for both EBV and HPVs in breast cancer; nevertheless, in our PCR data, co-incidence in breast cancer samples was 30%. Thirteen cases were both HPV and EBV negative.

IHC and PCR data for EBV and HPV are in good correlation (39% vs. 40% for EBV and 63% vs. 65% for HPV, respectively). However, since PCR is more sensitive as compared to IHC, data from PCR is considered reliable and was used for correlation analysis.

### Correlation of clinicopathological characteristics with HPV/EBV positivity

In our studied cohort, while we found HPV positivity not to correlate with tumor grade (*p* = 0.28), stage (*p* = 0.15) as well lymph node involvement (*p* = 0.08), EBV positivity correlated only with tumor grade (*p* = 0.02). In addition, the co-presence of high-risk HPVs and EBV (HPV + /EBV +) was associated with tumor grade (*p* = 0.03); nevertheless, there was no correlation with tumor stage (*p* = 0.81) and lymph node involvement (*p* = 0.15).

## Discussion

To our knowledge, this is the first study regarding the presence/co-presence of high-risk HPVs and EBV in human breast cancer and its association with tumor grade in the Lebanese population. Our data revealed that high-risk HPVs are present in 65% of breast cancer samples; while normal breast samples revealed positivity for high-risk HPVs in 5 of 14 cases; however, a significant difference is present in HPV-positivity between normal and tumor breast tissue samples (*p* = 0.04). Earlier studies have described the presence of high-risk HPVs in human breast cancer patients worldwide including the Middle East (ME) region, with prevalence varying from 4 to 86% [29, 35, 41, 44–47]. Explicitly, our results are in concordance with several reports in the ME region including Turkey, Qatar and Syria, where a high frequency of high-risk HPVs of 74%, 64% and 61%, respectively were reported [28, 29]. However, it is important to highlight that in the Lebanese population, HPVs prevalence ranges between 76 and 6% in different types of cancers such as oropharyngeal and cervical, respectively, with HPV16 being the most prominent [48, 49]. On the contrary, one of the major findings in our study is the predominance of HPV genotypes 52, 35 and 58 in breast cancer from Lebanese women (Fig. 1), which is similar to the Qatari population, where, HPV type 52 is the most prevalent subtype in breast cancer [35]. Moreover, HPV type 35 has been reported in breast cancer in Syrian and Turkish populations [28, 29]. Remarkably, studies in Bahraini and Omani women with cervical cancer, reported that HPV52 is the most prevalent subtype [50, 51]. While it is essential to point out that HPV types 16 and 18 are the most commonly found genotypes in cancers worldwide [45, 46]; in our study we did not detect HPV type 18, while HPV type 16 has a prevalence of 18% (Fig. 1). Similar to our data, HPV type 18 has a low prevalence in Syrian breast cancer samples [29]. However, analysis in breast cancer samples from Chinese population did not detect the presence of HPV types 16 and 18 [52]. Moreover, PCR analysis on breast cancer samples from Australia detected HPV type 18 as the most prevalent subtype in breast cancer specimens followed by HPV types 16, 45, 58 and 73 [53]. Therefore, this variation in the prevalence of HPV and genotype distribution is attributed to geographical locations, sample size as well as methodological differences, as it has been described previously [46, 54]. In addition, it is important to highlight that an earlier study from our group has shown that HPV type 16 is the most frequent in breast cancer in Canadian women [22]. Thus, our data corresponds with our previous work as well as other published studies related to the difference in the geographic distribution of HPV types in breast cancer. Moreover, it has been revealed that the presence of high-risk HPVs can be associated with cancer phenotype [9, 11, 28].

On the other hand, in human breast cancer, the presence of EBV is detected in almost 30–50% of cancer cases worldwide [55–59]. In this context, studies from Turkey and Syria reported 58% [60] and 52% [61] EBV positivity in breast cancer, while investigations from Egypt and Qatar revealed that EBV is present in 45% [62] and 49% [35] of breast cancer samples, respectively. In addition, the presence of EBV in breast cancer samples was also reported in Tunisia (27%) [63] and Iraq (28%) [62]. And recently, studies in breast cancer from Iran reported 27% of EBV DNA in their samples [64, 65]. In our study, we found that EBV is present in approximately 40% of breast cancer in Lebanese women. However, the underlying mechanism by which EBV infects mammary epithelial cells is still nascent. In this regard, a study by Hu et al. [66] showed that EBV infects mammary epithelial cells expressing CD21 and results in the growth of early mammary epithelial cells with a stem cell phenotype. EBV infection of the mammary epithelial cells alters gene expression (EBVness) and stimulates the oncogenic signaling via c-MET [66]. Moreover, EBV infection along with activated Ras triggers breast cancer development; EBV infection may prompt transformation of mammary epithelial cells to malignant cells, however, EBV is not vital once malignant transformation has transpired [66]. Therefore, our data indicate that the prevalence of EBV in breast cancer tissues in Lebanon is almost similar to its incidence worldwide including the ME region.

Interestingly, based on others and our previous works, co-presence of high-risk HPVs and EBV is involved in the onset and progression of various cancers including HN, colorectal, cervical as well as breast [29, 31, 33, 34, 67, 68]. In concordance, in our present investigation, we report that 29% of breast cancer cases, from Lebanese women, are co-infected with both HPVs and EBV. Our previous work in Syrian and Qatari samples pointed out that high-risk HPVs and EBV are co-present in 32% [34] and 47% [35] of breast cancer samples, respectively. Moreover, several investigations have demonstrated an association between the co-presence of HPVs and EBV and advanced breast Nottingham histological grade [55] as well as aggressive tumor phenotype [69]. Concurrently, we herein demonstrate in our examined cohort that the co-presence of high-risk HPVs and EBV has an association with advanced tumor grade (*p* = 0.03); indicating a plausible cooperative role of high-risk HPVs and EBV oncoproteins in the initiation and/or progression of various subtypes of human breast cancer as previously reported by several studies [55, 70–72]. Moreover, we recently pointed out that interaction of high-risk HPVs and EBV oncoproteins (E6/E7, LMP1 and EBNA1) can play an important role in the initiation and/or progression of human breast and oral carcinomas via the epithelial-mesenchymal transition (EMT) event [13, 73], which suggests an analogous mechanism in the pathogenesis of human breast malignancy. Based on the pathogenic role of HPVs and EBV in cancer, we revealed that oncoproteins of HPVs can interact with those of EBV resulting in cancer progression of different types of malignancies including HN, colorectal as well as breast, by the initiation and/or amplification of the EMT event [13, 31, 40, 73].

## Conclusions

This study clearly shows the co-presence of high-risk HPVs and EBV in Lebanese breast cancer women and implies that it is associated with tumor grade in our examined samples. However, we believe that more studies in a larger cohort from different countries of the ME region including Lebanon are necessary to ratify the presence/co-presence of HPV types and EBV in breast cancer and their association with tumor phenotypes. Furthermore, it would be of great interest to identify the cellular and molecular mechanisms that associate the co-presence of HPV and EBV infections with the initiation/progression of breast cancer to metastatic forms. Such investigations can explain the synergistic carcinogenic effect of those oncoviruses in breast cancer disease and verify if high-risk HPVs and future EBV vaccines could aid in preventing the development and/or progression of certain subtypes of breast cancer worldwide including the ME region. Moreover, it is important to take into consideration the most common HPV types in the region in order to select the appropriate HPV vaccine.

## Data Availability

All data generated or analyzed during this study are included in this published article [and its supplementary information files].

## Abbreviations

DCIS: Ductal carcinoma in-situ
EBER: Epstein–Barr encoding region
EBNA: Epstein–Barr virus nuclear antigen
EBV: Epstein–Barr virus
EBNA: EBV-encoded nuclear antigens
ER: Estrogen receptor
FFPE: Formalin-fixed paraffin embedded
H&E: Hematoxylin and eosin
HPV: Human Papilloma Virus
IDC: Invasive ductal carcinoma
IHC: Immunohistochemistry
ILC: Invasive lobular carcinoma
LMP: Latent membrane protein
PCR: Polymerase Chain Reaction
PI: Proliferative index
PR: Progesterone receptor
SPSS: Statistical Package for the Social Sciences
TMA: Tissue Microarray
